# Intracortical Inhibition in the Affected Hemisphere in Limb Amputation

**DOI:** 10.3389/fneur.2020.00720

**Published:** 2020-07-30

**Authors:** Ludmilla Candido Santos, Fernanda Gushken, Gabriela Morelli Gadotti, Bruna de Freitas Dias, Stella Marinelli Pedrini, Maria Eduarda Slhessarenko Fraife Barreto, Emanuela Zippo, Camila Bonin Pinto, Polyana Vulcano de Toledo Piza, Felipe Fregni

**Affiliations:** ^1^Laboratory of Neuromodulation & Center for Clinical Research Learning, Department of Physical Medicine and Rehabilitation, Harvard Medical School, Spaulding Rehabilitation Hospital, Boston, MA, United States; ^2^Faculdade Israelita de Ciências da Saúde, São Paulo, Brazil; ^3^Hospital Israelita Albert Einstein, São Paulo, Brazil

**Keywords:** short intracortical inhibition, long intracortical inhibition, phantom limb pain, transcranial magnetic stimulation, cortical silent period

## Abstract

Phantom limb pain (PLP) affects up to 80% of amputees. Despite the lack of consensus about the etiology and pathophysiology of phantom experiences, previous evidence pointed out the role of changes in motor cortex excitability as an important factor associated with amputation and PLP. In this systematic review, we investigated changes in intracortical inhibition as indexed by transcranial magnetic stimulation (TMS) in amputees and its relationship to pain. Four electronic databases were screened to identify studies using TMS to measure cortical inhibition, such as short intracortical inhibition (SICI), long intracortical inhibition (LICI) and cortical silent period (CSP). Seven articles were included and evaluated cortical excitability comparing the affected hemisphere with the non-affected hemisphere or with healthy controls. None of them correlated cortical disinhibition and clinical parameters, such as the presence or intensity of PLP. However, most studies showed decreased SICI in amputees affected hemisphere. These results highlight that although SICI seems to be changed in the affected hemisphere in amputees, most of the studies did not investigate its clinical correlation. Thus, the question of whether they are a valid diagnostic marker remains unanswered. Also, the results were highly variable for both measurements due to the heterogeneity of study designs and group comparisons in each study. Although these results underscore the role of inhibitory networks after amputation, more studies are needed to investigate the role of a decreased inhibitory drive in the motor cortex to the cause and maintenance of PLP.

## Introduction

Amputation is associated with significant cortical reorganization. After amputation, cortex's afferent inputs from amputated limb are interrupted, resulting in decreased cortical excitation, affecting local inhibitory drive (1). Decreased cortical inhibition seems to be maladaptive and can be related with other dysfunctional behaviors, like PLP (2, 3).

One approach to appraise maladaptive cortical reorganization is Transcranial Magnetic Stimulation (TMS), a non-invasive brain stimulation technique that induces small current resulted from magnetic fields, allowing assessment of both cortical excitability parameters and therapeutic modulation, inducing plasticity (4). TMS has been used to evaluate changes in cortical excitability following amputations. Different groups have shown changes in motor evoked potential (MEP), including decreased intracortical inhibition (ICI), cortical silent period (CSP), and increased intracortical facilitation (5).

TMS evaluates cortical excitability by different parameters: motor threshold, motor evoked potential, intracortical facilitation and intracortical inhibition (measured by intracortical inhibition, ICI, or cortical silent period, CSP). Short intracortical inhibition (SICI) and ICI are responses triggered when subthreshold stimulus is followed by a suprathreshold, within a range of 1–6 ms of interstimuli interval. Usually SICI is performed with a <5 ms interval and is thought to be related with GABAA receptors (6) while ICI with GABAB (7). LICI can be elicited by a subthreshold stimuli followed by a test stimulus with a 50–200 ms inter-interval (7). The mechanism is related to suppression of neuronal activity by GABA receptors. Lastly, CSP is defined as an interruption of electromyography activity following a suprathreshold TMS pulse (8), being related to GABA interneurons activation (9).

One study Cohen et al. (10) showed that amputees presented larger MEP in affected hemisphere and increased number of excitable stimulation sites for muscles proximal to the stump. However, few studies analyzed these parameters appropriately (11). Amputation studies are heterogeneous regarding amputated limb location, time since amputation and reimplantation. Investigation of maladaptive cortical reorganization could contribute to developing novel treatments for PLP.

Phantom pain and sensations affect up to 98% of amputees (12). PLP is the most prevalent phantom phenomena (50–80%) (11), with negative impact on quality of life (11, 13). Studies suggest that cortical reorganization is reversible and related to pain levels (14), sheding light on the potential minimization or reversal of maladaptive plasticity through brain stimulation. However, most articles do not correlate changes in excitability with presence or intensity of PLP, implicating that changes in these parameters have unknown mechanisms.

Despite no consensus about etiology and pathophysiology of phantom experiences, studies associate PLP with peripheral, psychogenic, and central neural mechanisms (15, 16) and with cortical reorganization after an amputation (17, 18). Other studies show that peripheral systems contribute to neuromas' formation, followed by hyperexcitability and spontaneous discharges (19), while psychological systems may influence its intensity (20). Different patterns of change were observed in amputees with or without PLP: (1) decreased ICI in affected hemisphere; (2) decreased ICI in non-affected hemisphere; (3) unchanged ICI in affected hemisphere; and (4) changes in CSP response pattern.

Therefore, the purpose of this review is to evaluate if CSP and SICI are modified when comparing the affected vs. unaffected motor cortex and whether it provides additional insights to the role of motor cortex in the modulatory circuitry of chronic pain.

## Materials and Methods

### Sources and Study Selection

Literature search was performed in four electronic databases (PubMed, Web of Science, ScienceDirect, and LILACS) until February 2018, using multiple keywords and combinations—“phantom limb” AND “neuromodulation” OR “transcranial magnetic stimulation” OR “cortical excitability” OR “neuronal plasticity.” The conjunction “phantom limb AND transcranial magnetic stimulation” was combined with “intracortical inhibition” OR “cortical silent period” OR “neuromodulation.” Initial search identified 2,284 articles.

Pairs of researchers analyzed selection criteria and a third person resolved conflicts. Included articles had to: (1) be related to amputation; (2) evaluate phantom sensation; (3) use TMS as an assessment tool; (4) have data on ICI or CSP. Studies were excluded if: (a) not related to amputation; (b) related to congenitally absent limbs; (c) just included finger amputation; (d) applied different techniques of stimulation as DBS, spinal cord stimulation, tDCS, TENS, fMRI; (e) applied TMS for cortical mapping; (f) did not use neurostimulation; (g) had only pharmacologic interventions; (h) had solely psychotherapeutic approaches; (i) analyzed mirror therapy not combined with TMS/TDCS; (j) had different studies designs as posters, reviews or meta-analysis; (k) were not in English; (l) studies in animals.

The selected articles were inputted into COVIDENCE® software, which excluded duplicates, resulting in 1313 articles. Three articles were included in a manual search due to discussion of cortical excitability using TMS in amputees with PLP (21).

## Results

### Studies Selection

After screening titles and abstracts, 42 articles remained. After full text reading, seven articles were then selected.

### Demographic Findings

One hundred and eighteen patients were analyzed, healthy subjects (45) and amputees (73). Most amputees were male young adults with traumatic upper limb amputation (5, 22–24). Six articles reported PLP (5, 22–26). Sample size, population profile, measurements, comparisons, and etiology of amputation were diverse (Table 1).

**Table 1:** General characteristics of selected papers.

**Author**	**Type of study**	**Sample size (Amputees/controls)**	**Intervention**	**Assessment**	**Comparison**	**Reported ICI measurement**	**Results Cortical Inhibition**	**Results CSP**	**Level of amputation**	**Etiology of amputation**	**TMS coil type**	**ISI (ms)**	**Muscles for surface EMG**
Bestmann et al. (24)	Case report	1 (1/0)	N/A	TMS during fMRI	N/A	ICI (2–3 ms ISI) (%)	Left FDI 45%; Left Del 68%; Right Del 84%	Left FDI: 124 ms; Left Del: 95 ms; Right Del 112 ms	Right arm amputee	Traumatic injury	Figure of eight	2–3 (pool)	Deltoid and FDI
Chen et al. (27)	Cross-sectional	23 (16/7)	N/A	TMS	Healthy vs. affected hemispheres; Healthy controls vs. amputees	MEP at Inhibitory ISIs 2–4 ms (%)	MEP amplitude on the amputated side (240 ± 121% of control) was significantly larger compared with the intact side (60.1 ± 7.6%) and with normal subjects (59.6 ± 7.5%)	N/A	Lower limb amputees	Traumatic injury, tumor, diabetes/vascular, infectious causes	Circular	2 and 4 (average)	Quadriceps
Dettmers et al. (5)	Case report	1 (1/0)	N/A	TMS, fMRI	Healthy vs. affected hemispheres; Healthy controls vs. amputees	N/A	Reduction of ICI (no numerical data)	Aa: 110.1 ms; Naa: 142.0 ms	Upper limb amputees	Traumatic injury	Figure of eight	1–4 (individual values)	Deltoid muscle
Fitzgibbon et al. (25)	Cross-sectional	25 (14/11)	N/A	TMS	Healthy vs. affected hemispheres; Healthy vs. mirror pain vs. no mirror pain	Mean SICI	**Controls:** SICI LH 34.5 (17.38), SICI RH 44.04 (26.32); **Mirror Pain +:** SICI LH 40.92 (22.80), SICI RH 45.05 (18.20); **Mirror Pain -:** SICI LH 57.77 (47.54), SICI RH 36.92 (16.59)	N/A	Lower limb amputees	Traumatic injury, tumor, diabetes/vascular	Figure of eight	2	FDI
Hordacre et al. (26)	Case control	26 (13/13)	N/A	TMS	Healthy vs. affected hemispheres; Healthy controls vs. amputees	Laterality index LI^*^ (mean, SD)	**SICI**: M1CON Control 0.86 (0.1), AA 0.91 (0.1), AD 0.79 (0.2); M1IPSI: Control 0.89 (0.1), AA 1.03 (0.1), AD 0.82 (0.2); **LICI**: M1CON Control 0.64 (0.3), AA 0.70 (0.3), AD 0.61 (0.4); M1IPSI: Control 0.73 (0.3), AA 0.73 (0.2), AD 0.69 (0.3)	N/A	Unilateral transtibial amputees	Did not provide this information	Figure of eight	2	Quadriceps
Schwenkreis et al. (22)	Cross-sectional	18 (12/6)	N/A	TMS	Healthy vs. affected hemispheres; Healthy controls vs. amputees	Averages of MEP ratios obtained at inhibitory interstimulus intervals of 1 ± 5 ms	UAA: 42.7 ± 19.8%; UANAS 31.9 ± 17.8%; FA: 69.9 ± 16.5%; FNAS 47.7 ± 14.1%	UAA: 58.3 ± 22.9 ms; UANAS: 76.4 ± 20.1 ms; FA: 111.5 ± 38.2 ms; FNAS: 117.1 ± 38.8 ms	Upper limb amputees	Traumatic injury, tumor	Circular	1–5 (pool)	Deltoid or biceps
Schwenkreis et al. (23)	Part of RCT	24 (16/8)	Memantine	TMS	Healthy controls vs. amputees; Placebo vs. memantine	Averages of MEP ratios obtained at inhibitory interstimulus intervals of 1 ± 5 ms	**MG:** baseline 51.8% (41.0–105.0), day 21 43.2% (11.0–77.0) **PG:** baseline 51.6% (14.0–116.0), day 21 47.7% (15.0–138.0) **Control:** baseline 20.9% (10.0–36.0)	N/A	Upper limb amputees	Traumatic injury, tumor	Circular	1–5 (average)	Deltoid, biceps, or FDI

*Left FDI, Left first dorsal interosseous; Left Del, Left Deltoid; Right Del, Right Deltoid; Aa, affected arm; Na, non affected arm; LH, left hemisphere; RH, right hemisphere; AA, amputee admission; AD, amputee discharge; UAA, upper arm amputation affected side; UANAS, upper arm amputation non affected side; FA, forearm amputation affected side; FNAS, forearm amputation non affected side; MG, memantine group; PG, placebo group; w, week*.

* *LI: (MEP amplitudeM1CON – MEP amplitudeM1IPSI)*.

*(MEP amplitude M1CON + MEP amplitude M1IPSI)*.

*M1, motor cortex; CON, contralateral; IPSI, ipsilateral*.

### Study Design

From the studies selected, there were three cross-sectional (22, 25, 27), two case reports (5, 24), one part of a clinical trial (23), and one case-control (26). Regarding technical aspects, some (5, 24–26) used a figure-of-eight-coil, while others (22, 23, 27), a circular coil. The muscles chosen for surface electromyography were deltoid (5, 22–24), biceps (22, 23), first dorsal interosseous (FDI) (23–25), and quadriceps (26, 27) (Table 1).

Five articles used TMS as assessment tool for cortical excitability: one study (5) performed functional MRI and TMS and four studies used TMS only (22, 25–27). Furthermore, one article (23) added pharmacological intervention (memantine) and another (24) applied TMS-fMRI as intervention.

### Qualitative Analysis of the Studies

Single and paired-pulse TMS were applied to investigate SICI; however, paired-pulse TMS assessment protocols were highly heterogeneous (Table 1). Conventionally (28), subthreshold conditioning stimulus (usually at 80% of the motor threshold) is followed by suprathreshold test stimulus at interstimulus intervals (ISIs) of 1–5 ms. Therefore, SICI is measured by the reduction of relative MEP amplitude by subthreshold conditioning stimuli, compared to average MEP size. In this review, ISIs varied from 1 to 5 ms and were reported separately or as intervals average.

The comparison types of SICI varied (Table 1). Some compared non affected with affected hemispheres (5, 22, 24, 26, 27), others compared amputees with healthy controls (22, 23, 25–27). However, Schwenkreis et al. (23) evaluated SICI measures before and after treatment (memantine vs. placebo), and Bestmann et al. (24) contrasted right with left deltoid, and also with FDI muscle independent of stimulation side. All CSP values were measured for both non-affected and affected hemispheres.

### Studies Showing Decreased ICI in the Affected Hemisphere

Regarding SICI findings, three studies (5, 22, 24, 27) compared amputee's SICI in affected vs. non-affected hemispheres, verifying a larger conditioned MEP amplitude in affected hemisphere (lower SICI).

**Figure 1:**
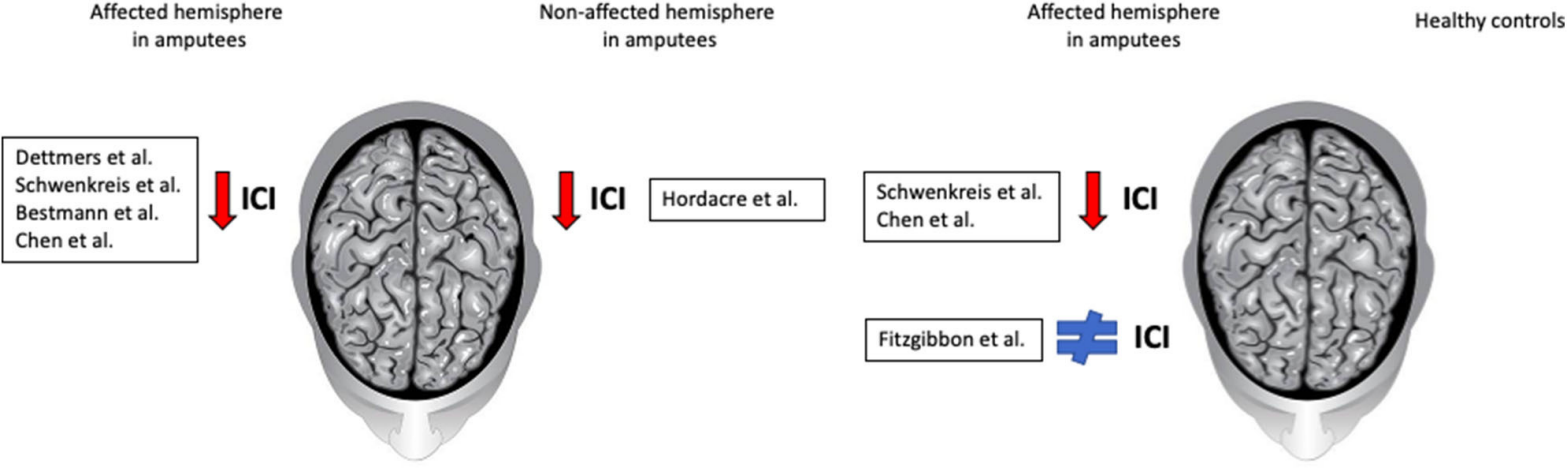
Summary results and comparisons of intracortical inhibition. The first panel shows comparisons between affected and non-affected hemisphere in amputees; most authors reported a decreased SICI in the affected hemisphere. The second panel shows the comparison between amputees and healthy subjects; while some authors report a decreased SICI in the affected hemisphere of amputees, others found no difference.

Both Schwenkreis studies (22, 23) reported a significant decreased SICI response in affected side compared to controls (Figure 1).

### Studies Showing Decreased ICI in the Non-affected Hemisphere

Only Hordacre et al. (26) found reduced SICI response in non-affected hemisphere (Figure 1). He assessed amputees's SICI at four time points: admission, prosthetic casting, first walk and discharge, and contrasted with healthy controls' SICI at admission. The mean SICI response was reduced in amputees' non-affected hemisphere compared to controls.

### Studies Showing Unchanged ICI in the Affected Hemisphere

Concerning SICI, while Schwenkreis et al. (22) found no differences between affected and non-affected hemispheres within each participant, Fitzgibbon et al. (25) found no differences between amputees and healthy controls' SICI (Figure 1). Subgroup analysis within amputees showed no differences between patients regardless of mirror pain. Chen et al. (27) found that mean MEP amplitude was significantly larger in affected hemisphere, compared with healthy controls.

### Changes in CSP Response Pattern

Regarding CSP, three articles (5, 22, 24) measured it once and compared affected with non-affected hemispheres (Table 1). While Schwenkreis et al. (22) found similar CSP measurements for both hemispheres, Bestmann et al. (24) suggested it was markedly longer when recorded at the affected side and finally Dettmers et al. (5) showed a shortened silent period on the affected side. Only Schwenkreis et al. (22) compared CSP between amputees and healthy controls and found no statistically significant differences.

## Discussion

In this review, we evaluated cortical excitability changes following upper/lower limb amputation to investigate whether it helps understand the cortical mechanisms associated with PLP development. Both measurements can be assessed by TMS and were investigated due to their role in cortical motor functioning. Most studies (5, 22, 24, 27) found a decreased mean SICI in the affected hemisphere compared with non-affected hemisphere and healthy controls. CSP results were inconclusive, especially because of scarcity of data. However, contrary to what was initially expected, most studies did not show any correlation of cortical excitability changes with presence or intensity of pain.

Central structural and function brain changes are described in several chronic pain conditions (29–32); while some are thought to be consequences of pain (29), others are not well-characterized. Regarding PLP, these alterations have gained more attention because different imaging studies have showed that cortical and plastic changes are involved with the presence of pain. In fact, these changes have opposite direction—while some showed PLP correlation with strong motor cortex reorganization and the missing representation of the amputated area (18, 33–35), others showed that it is actually correlated with the maintenance of amputated area representation (3). Despite the direction, all seem to agree on a maladaptive reorganization of the sensorimotor cortex after amputation involving a reduction in ICI mechanisms, an imbalance between inhibitory and excitatory neurotransmitters, and increased excitability of corticospinal neurons (36). Nonetheless, this reorganization does not seem to be related to pain intensity, being therefore a consequence or a cause of pain; but not related if the intensity perception or only a response to the deafferentation process occurring in amputees.

### Studies Showing Decreased ICI in the Affected Hemisphere

After amputation, the motor cortex undergoes modifications previously associated with the presence or intensity of PLP. However, reorganization is observed in amputees with Lotze et al. (35) and without pain, or with other chronic pain syndromes (37). Although mechanisms that lead to pain after amputation remains unknown, some TMS studies showed that changes in cortical excitability are frequently observed in amputees that experience PLP (10). They early found excitability enhancement in amputee's affected hemisphere due to larger MEPs and increased number of excitable stimulation sites, when compared with the intact limb (10). Moreover, studies using techniques such as functional magnetic resonance (fMRI) and positron emission tomography (PET) demonstrated larger blood-oxygen-level-dependent (BOLD) activity in the affected hemisphere of amputees with PLP, compared with amputees without PLP (38). This data suggests lack of affected hemisphere's inhibitory function after amputation. Mechanistic studies showed that in early phases after amputation, motor cortex reorganization is partially driven by downregulation of GABA-related inhibitory circuits (39), which also contributes to increased excitability observed then. Indeed lack of sensory afference likely drives changes that decrease inhibitory drive in cortical circuits, and also in pain-related circuits, resulting in PLP. Accordingly, SICI can be used to measure intracortical circuits within the motor cortex and is an indirect measure of GABA-mediated inhibition. Altered SICI can modify motor outputs and cortical-subcortical connectivity. A recent meta-analysis (37) showed a significant SICI reduction in patients with chronic pain when compared with healthy subjects, possibly relating to pain chronicity.

These data indicate motor cortex disinhibition in amputees' affected hemisphere, remaining unclear its relation with pain. Therefore, rather than predicting pain intensity, decreased inhibitory drive may relate to its presence according to studies that compared amputees against healthy subjects, however does not detangle amputation as its leading factor.

### Studies Showing Decreased ICI in the Non-affected Hemisphere

Longitudinal studies aiming to report changes in corticomotor excitability pre and post-amputation are uncommon. Hordacre et al. (26) compared motor cortex excitability before and after transtibial elective amputation and observed SICI reduction in both hemispheres after amputation (40). Whereas, decreased amputee's SICI agrees with our findings, most reviewed studies showed a reduction in the affected hemisphere, not bilaterally. The assessment shortly after an amputation allowed Hordacre et al. (26) to document the early modulation of intracortical excitability. This could indicate that cortical environment at this period is optimized for reorganization, representing potential timeframe favorable to successful interventions.

### Studies Showing Unchanged ICI in the Affected Hemisphere

Most studies (5, 22, 24, 27) showed decreased mean SICI in affected hemisphere, but Fitzgibbon et al. (25) showed no difference in SICI comparing amputees with healthy controls, and amputee's cortical excitability with and without mirror pain with no difference to healthy controls. The authors concluded that cortical disinhibition seems to be disassociated with mirror pain. However, acquired mirror pain likely has different mechanisms (2) compared to PLP.

### Changes in CSP Response Pattern

Mixed results were observed in three manuscripts (5, 22, 24) that compared affected vs. non-affected hemispheres on amputees, with similar findings for both groups (amputees vs. healthy controls. Future studies on CSP can elucidate ICI mechanisms in amputated patients.

Current evidence supports CSP association with GABA interneurons activation (37, 41), hypothesizing that irregular GABA activation could be monitored during increased CSP values in the affected hemisphere. Analyzing CSP alone could bring ambiguous results; the silent period cannot predict the motor cortex excitatory state, as other variables change simultaneously in a dynamic pattern. More extensive trials focused on CSP could address the issue of heterogeneity of study designs, sample size, and parameters. Then, it would be possible to analyze its applicability, investigating its potential use to tailor therapies focused on cortical activity and neuroplasticity, rather than only treating effects.

The persistent cortical representation of the missing limb and reassignment of brain areas, may explain why mirror therapy (42) is known as a promising tool for PLP management: volitive activation of cortex area of the phantom limb allows modulation and decreases thalamic processing (12, 15, 43). Mirror therapy studies showed that pain relief may be due to neuron firing when a person performs actions with the contralateral limb or observes someone's movements (44). Some studies correlated PLP severity and location with the onset of pain relief, indicating that more severe and intense types of PLP take longer periods to respond to mirror therapy. Still, patient variability and pain subtypes might interfere on the efficacy of mirror therapy (33, 45).

There is evidence that magnitude of cortical reorganization is associated with pain severity, and that the extent of somatosensory cortex involvement is related to intensity of phantom limb experience (46, 47).

### Limitations

This review included a limited amount of studies (7), composed of small samples (1 to 25) and a total of 118 individuals. Heterogeneity of parameters was concerning, as authors diverged on concept definitions for SICI, LICI, and CSP. Moreover, three of eight selected studies (22, 23, 27) used circular coil in TMS, which is not used anymore, thus not comparable to current studies.

These considerations emphasize the importance of leading research on biomarkers for PLP, so its underlying mechanisms could be better understood. These tools would enable follow up of patients' progression and allow individualized treatments, potentially decreasing the condition's burden.

## Conclusion

In conclusion, the SICI changes in amputees' affected motor cortex demonstrate lack of inhibitory stimuli, suggesting it could be a useful marker to understand the consequences of amputation. However, none of the studies were able to associate this finding with clinical correlates; thus additional studies would be worthwhile to answer this question. Regardless, we showed combined evidence that amputees have decreased cortical inhibition in the affected motor cortex. Future studies evaluating differences in SICI and CSP between amputees with and without pain could provide new insights regarding maladaptive changes occurring after limb amputation and its relationship with PLP.

## Author Contributions

FF and CP contributed as senior authors, aiding in the study design, and conceptual ideas. BD, EZ, FG, GG, LC, MB, PP, and SM contributed equally in the manuscript writing, design of figures, and data analysis. All authors contributed to the article and approved the submitted version.

## Conflict of Interest

The authors declare that the research was conducted in the absence of any commercial or financial relationships that could be construed as a potential conflict of interest.
